# An Investigation of Signal Preprocessing for Photoacoustic Tomography

**DOI:** 10.3390/s23010510

**Published:** 2023-01-02

**Authors:** Isaac Huen, Ruochong Zhang, Renzhe Bi, Xiuting Li, Mohesh Moothanchery, Malini Olivo

**Affiliations:** Institute of Bioengineering and Bioimaging, A*STAR, 11 Biopolis Way, Singapore 639798, Singapore

**Keywords:** photoacoustic tomography, preprocessing, wavelet denoising, CNR, resolution

## Abstract

Photoacoustic tomography (PAT) is increasingly being used for high-resolution biological imaging at depth. Signal-to-noise ratios and resolution are the main factors that determine image quality. Various reconstruction algorithms have been proposed and applied to reduce noise and enhance resolution, but the efficacy of signal preprocessing methods which also affect image quality, are seldom discussed. We, therefore, compared common preprocessing techniques, namely bandpass filters, wavelet denoising, empirical mode decomposition, and singular value decomposition. Each was compared with and without accounting for sensor directivity. The denoising performance was evaluated with the contrast-to-noise ratio (CNR), and the resolution was calculated as the full width at half maximum (FWHM) in both the lateral and axial directions. In the phantom experiment, counting in directivity was found to significantly reduce noise, outperforming other methods. Irrespective of directivity, the best performing methods for denoising were bandpass, unfiltered, SVD, wavelet, and EMD, in that order. Only bandpass filtering consistently yielded improvements. Significant improvements in the lateral resolution were observed using directivity in two out of three acquisitions. This study investigated the advantages and disadvantages of different preprocessing methods and may help to determine better practices in PAT reconstruction.

## 1. Introduction

Photoacoustic or optoacoustic tomography (PAT) is an emerging modality that combines the high contrast of optical methods and good resolution in deep tissue with acoustic detection. Being able to break through the optical diffusion limit, PAT has shown great potential in the noninvasive detection of early-stage cancer [1] and the imaging of the brain [2]. When tissue is illuminated with a modulated light source, it absorbs energy followed by periodic thermal expansion to generate an ultrasonic wave. A transducer is employed to collect the propagated ultrasonic wave, which forms the final image by reconstruction. The signal-to-noise ratio (SNR) of the received PA signal is limited by several factors. First of all, the efficiency at each stage of energy conversion is not 100%, accompanied by additional thermal noise, electronic noise, etc. Moreover, in PAT, a high-power nanosecond pulsed laser is usually adopted to illuminate an area of tissue, and the amplitude of the generated PA signal in MHz is largely dependent on the optical absorption of the target. However, according to ANSI standards [3], there is a maximum permissible exposure (MPE) of lasers on human skin, which limits the excitation energy delivered into tissue and the strength of the generated signal. Because of these factors, the received PA signal could be weak and noisy, which may affect the final image quality, especially in deep tissue. 

There are several preprocessing methods to enhance the raw PA signal before reconstruction. Excluded from this consideration is the averaging of multiple acquisitions, as increased acquisition time may affect the imaging of tissue motion. A signal is typically decomposed into components (the basis functions characterized by coefficients), with differences between the signal and noise coefficients allowing for the removal of noise. The Fourier transform method uses a simple basic function, the sinusoid, characterized by its frequency [4]. Hence lowpass, bandpass, and matched filters exclude noise-dominated frequencies, although this could also result in a signal loss if it occupies the same frequencies [5,6].

Improved results could be obtained by changing the basis function to waveforms matched to the signal, as in wavelet denoising [7,8]. The signal is decomposed using the discrete wavelet transform method into wavelet coefficients where, ideally, the signal and noise are separated. These noise coefficients are then removed based on thresholds, and the pure signal is reconstructed using the inverse wavelet transform method. This approach was demonstrated to yield SNR improvements in many applications, including chick embryos and rat brains [9], as well as in photoacoustic microscopy in melanoma cells [10]. It was shown to outperform both lowpass and bandpass filters, with bipolar “Symlet” family wavelets found most effective both in silico and in vivo, although the parameters depended on the frequency content and SNR of the image [7].

Empirical mode decomposition (EMD) sifts (decomposes) signals into intrinsic mode functions (IMFs), which have the advantage of being able to vary in frequency and amplitude. Feature selection is then used to identify noisy IMFs and remove them. This was previously demonstrated to improve photoacoustic images in several studies, including a simple approach of merely including the first two IMFs [11,12,13].

Singular value decomposition (SVD) was also used to remove laser-induced noise in photoacoustic images [11]. This is based on decomposing the imaging operator *H,* which is responsible for noise, into its component matrices USV^T^, where S is a diagonal matrix containing the singular values of each component. Noisier components could then be removed; indeed, better performance was achieved using just a single component [10].

It is also of relevance that sensors are not omnidirectional but less sensitive in particular directions [14]. The noise-related artefacts resulting from this were illustrated with simulations to date, including using the MATLAB k-wave toolbox [15] and Monte Carlo simulation of red blood cell signals, demonstrating a fourfold increase in accuracy when reconstruction accounted for sensor directivity, according to [16]. 

However, to our knowledge, there has not been a detailed comparison of the combinations of common preprocessing methods, and thus an optimal preprocessing workflow has not yet been defined. Non-classical methods, such as deep learning, were demonstrated for noise reduction, object detection, and segmentation [17]. However, these are not suitable for inclusion in the comparison because they are not a well-defined algorithm but are trained to datasets, which are typically simulated with limited translatability to experimental datasets [18]. We, therefore, restricted the scope of comparison to common classical methods.

In this paper, we aimed to investigate and optimize the preprocessing of PA signals from a 256-element multi-segment transducer [19] by comparing the reconstructed 2D PA image quality of the combinations of the unfiltered signal, bandpass filter, deconvolution, wavelet denoising, and sensor directivity. We used the metrics of noise reduction (measured as the contrast-to-noise ratio [CNR]) and resolution in both directions (measured as FWHM) to quantify the performance of each combination. These were investigated in a range of acquisitions from single-point sources, to multiple-point sources and finger imaging.

## 2. Materials and Methods

### 2.1. Image Acquisition

**Figure 1:**
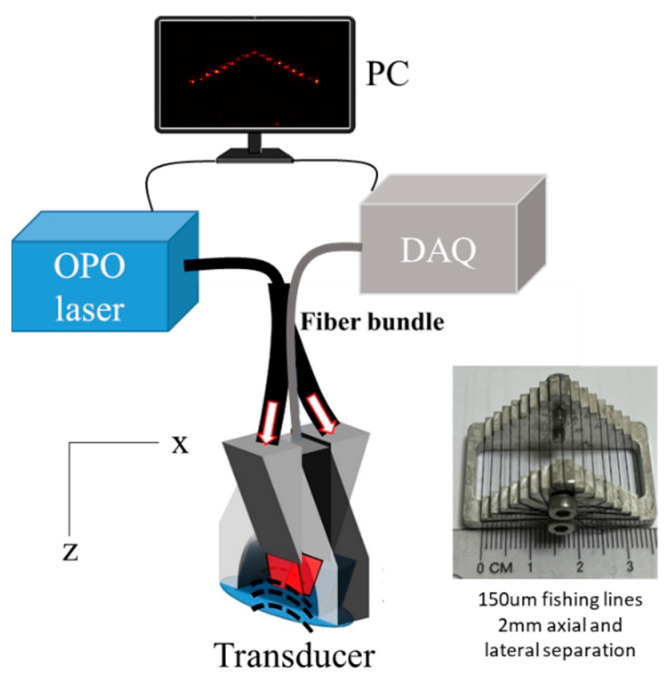
Schematic of the photoacoustic imaging system and the phantom. A total of 17 150 µm-fishing-lines were arranged in a pyramid shape with a metal frame. The axial and lateral separations were 2 mm between the two lines. Cross-sectional imaging was performed from the top of the phantom. OPO: optical parametric oscillator. DAQ: data acquisition unit.

A 20 Hz tunable optical parametric oscillator (OPO, Ekspla) nanosecond pulsed laser was shone through a fiber bundle from one side of the transducer to illuminate the sample. The output energy at 700 nm was fixed at 45 mJ with a 3 cm^2^ illumination area, corresponding to a fluence of ~15 mJ/cm^2^. A customized multi-segment transducer with 256 elements arranged in an arc-linear-arc shape was used to acquire the PA data, as described in [19]. The sampling rate of the data acquisition unit (DAQ) was set to 40 MHz, and 3072 points were acquired for each channel. A PC was used to control the software written in MATLAB and save the data. In the phantom experiments, a 150 µm black fishing line was imaged first (“point” acquisition). A phantom composed of 17 fishing lines arranged in a pyramid shape was also used, as shown in Figure 1 (“multi-point” acquisition). This consisted of 1 point at the top, with 8 rows of increasing lateral separation below. The axial and lateral separation between successive points was 2 mm. Moreover, imaging on a finger was also performed (“finger acquisition”). 

### 2.2. Signal Processing

**Figure 2:**
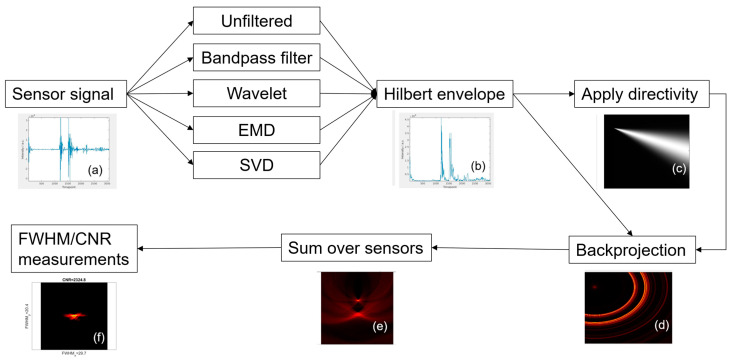
Example of a raw signal from the single sensor (**a**) followed by a Hilbert envelope (**b**). Angular intensity of optional directivity (**c**). Backprojection of a single sensor (**d**) followed by summing over all sensors (**e**). FWHM and CNR measurements (**f**).

All image processing was carried out in MATLAB R2019b. The signal processing workflow for all compared methods is shown in Figure 2. The parameters for all methods are shown in Table 1.

The sensor signal underwent a bandpass filter, wavelet denoising, EMD, or SVD, or was unfiltered, followed by applying directivity or not. This yielded 10 combinations (unfiltered, bandpass, wavelet, EMD, SVD, unfiltered+directivity, bandpass+directivity, wavelet+directivity, EMD+directivity, SVD+directivity).

#### 2.2.1. Optimization of Preprocessing Methods’ Parameters

The bandpass filter’s FWHM was set at 90% of the central frequency.

The wavelet denoising parameters (wavelet family, order, denoising method, threshold rule, and noise estimation) were optimized with CNR maximization and the minimization of FWHM_x_ and FWHM_z_ (Appendix A). It was found that the wavelets of the Daubechies family performed best in the fourth order. The optimized values of all parameters are listed in Table 1. 

EMD was performed using the MATLAB emd function by iteratively decomposing the data X(t) into IMFs and a residual. This involved the following steps [20,21]:

1. Finding all local minima and maxima of the data;
2. The calculation of the lower and upper envelopes by the cubic spline interpolation of the minima and maxima, respectively;
3. Calculating the mean of the lower and upper envelopes, $m_{1}$;
4. Calculating the provisional component *h_1_* as the difference between the data and $m_{1}$: $h_{1}=X\left(t\right)-m_{1}$;
5. Repeat sifting (steps 1–4) but using the provisional component as the data, yielding $h_{11}=h_{1}-m_{11}$;
6. Continue sifting *k* times until the stopping criterion is reached, yielding $h_{1k}=h_{1\left(k-1\right)}-m_{1}$. This is the first component, $c_{1}$;
7. Derive the residue $r_{1}$: $r_{1}=X\left(t\right)-c_{1}$;
8. Repeat steps 1–8 using the residue *r_1_* as the data, yielding $c_{2}=r_{1}-r_{2}$;
9. Continue repeating steps 1–8 to yield all further components: $c_{n}=r_{n-1}-r_{n}$;
10. Stop when the residue $r_{n}$ is a monotonic function, meaning no more minima and maxima exist, and thus no more components are extracted.

This was followed by summing the first two components and taking their absolute value.

SVD was performed using the MATLAB svd function to decompose the data matrix *X* of dimension *n* × *p* (where *n* is the number of sensors and *p* is the number of time points) into a matrix of left vectors U of the dimension *n* × *n*, a diagonal singular value matrix S of the dimension *n* × *p*, and a matrix of right vectors *V* of the dimension *p* × *p*.

$$X=USV^{T}\;$$

As previously demonstrated, this decomposition characterized the laser-induced noise in particular because the noise was consistent across sensors, and using one singular value component was sufficient [10]. Therefore, all values in *S* except the first diagonal value were set to zero to form the laser-induced singular value matrix *S_L_*. The noise was then calculated as *US_L_V^T^* and subtracted from the original signal *X*.

After these preprocessing steps were completed, the universal back-projection algorithm [22] was used for image reconstruction. 

#### 2.2.2. CNR Measurement

CNR could be defined in the following way [23]:

CNR=|S_I_−S_o_|/σ_o_ (1)

where S_I_ and S_o_ are the means of the ROIs located inside and outside the signal of interest, respectively, and σ_o_ is the standard deviation of the ROI located outside.

A signal ROI of 20-pixel half-width was drawn centered on the pixel with the maximum signal. S_I_ was thus the mean of this region. A noise ROI of 50-pixel half-width was drawn in the top left of the image, away from the signal or artefact. The S_noise_ and σ_noise_ were the mean and standard deviation of this ROI, respectively. We then calculated the CNR = |S_I_−S_noise_|/σ_noise_.

As another indicator of image quality, we also calculated the peak SNR where:

Peak SNR= S_Imax_/σ_o_ (2)

where S_Imax_ is the maximum value of the signal ROI, and σ_o_ is the standard deviation of the noise ROI.

#### 2.2.3. Resolution Measurement

FWHM_x_ and FWHM_z_ were used to represent the lateral and axial resolution of the reconstructed image. The FWHM_x_ measurements were performed inside the signal ROI centered on the pixel with the maximum signal identified in 2.2.2. The signal was taken from a range of x-coordinates 20 pixels below and above this pixel, yielding 41 points in total. Its minimum was subtracted to eliminate the need to fit a constant as an additional parameter. This was then fitted to a three-term Lorentzian function (using the *lorentzfit* function from MATLAB Central) to yield its scale parameter γ. This was multiplied by 2 to yield FWHM_x_. The analogous procedure was repeated in the z-direction to yield FWHM_z_. Since these were in units of pixels, they were converted to μm via multiplication by the x- and z-resolutions.

The multi-point acquisition had a range of intensities at different points, and we, therefore, needed to select a single measurement for comparison. We compared the CNR and resolution measurements for every point using the default unfiltered method (Appendix B). The best results were obtained in the middle row (Appendix B), and thus, the CNR and resolution results were reported as the average of the pair of points in this row.

The results were reported as CNR, FWHM_x_, and FWHM_z_. The changes relative to the control (unfiltered) method were reported as ΔCNR, ΔFWHM_x_, and ΔFWHM_z_.

## 3. Results

**Figure 3:**
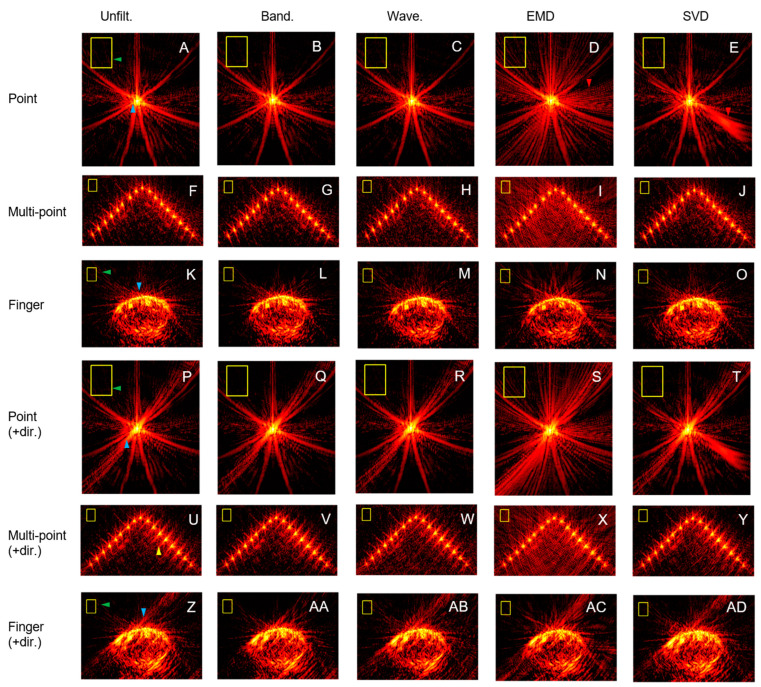
Whole images shown on the log scale widened to show the noise for all methods for the point, multi-point, and finger acquisitions without directivity (first three rows) and with directivity (last three rows). Noise ROIs were annotated as yellow boxes. The dynamic range was 50 dB for point acquisition, 40 dB for multi-point acquisition, and 35 dB for finger acquisition. The markers show the points of comparison mentioned in the discussion.

Whole images are shown, for example, the methods in the point, multi-point, and finger acquisitions (Figure 3) with the noise ROI annotated.

**Figure 4:**
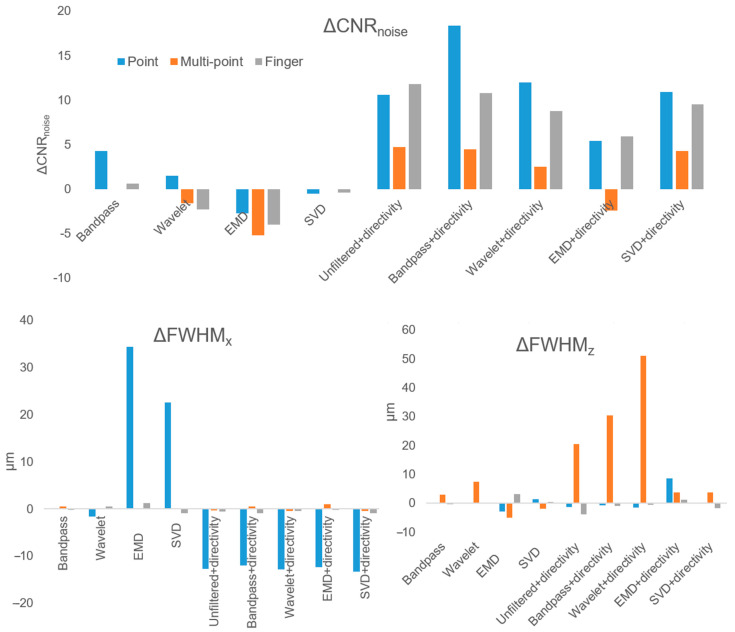
Comparison of the changes in CNR, FWHM_x_, and FWHM_z_ from the unfiltered method.

The CNR and FWHM measurements are shown in Table 2. A higher CNR represents better noise reduction, while a lower FWHM represents better resolution. Comparisons with the actual imaging phantom were listed in the brackets under the FWHM columns. The changes relative to the unfiltered method were calculated for all measurements (ΔCNR, ΔFWHM_x_, and ΔFWHM_z_) in Figure 4.

The unfiltered+directivity method had the highest ΔCNR in two of three acquisitions, with bandpass+directivity being second and highest in the other. Averaged across all three acquisitions, bandpass+directivity had the highest ΔCNR, unfiltered+directivity was second, SVD+directivity was third, wavelet+directivity was fourth, and EMD+directivity was last. For the non-directivity methods, the same order resulted. The CNR of the directivity methods was significantly higher than the non-directivity methods (paired t-test, *p* < 0.05) for all three acquisitions. 

There were various trends for FWHM. The five largest FWHM_x_ decreases occurred using the five directivity methods in the point acquisition. In the multi-point acquisition, five of the six largest FWHM_z_ increases were from the directivity methods. The FWHM_x_ of the directivity methods was significantly lower than the non-directivity methods (paired t-test, *p* < 0.05) in all acquisitions except for the multi-point acquisition.

## 4. Discussion

The use of directivity significantly improved noise reduction in all acquisitions. Though it significantly improved lateral resolution in two of the acquisitions, its effects on the resolution were mixed. There appeared to be two main effects of directivity: reductions in the intensity of the streaks broadly from the top-left to the bottom-right direction (green markers, Figure 3A vs 3P, Figure 3K vs 3Z), which is closest to the lateral direction and thus likely measured as an improvement of lateral resolution. They also reduced the noise (measured in the yellow box), resulting in the observed increase in the CNR. There are also increases in the streak length broadly from the top-right to the bottom-left direction (blue markers, Figure 3A vs 3P, Figure 3K vs 3Z), which is closest to the axial direction and thus likely measured as a worsening of axial resolution. The magnitude of the measured effects depended on how lateral the signal ROI was from the sensor array. This was relatively central in the point and finger acquisitions but more lateral in the multi-point acquisition because it was in the middle row. Considerable variations in streak lengths are seen at different locations using directivity in the multi-point acquisition (yellow marker, Figure 3U), which may be why the multi-point acquisition lacked a significant improvement in lateral resolution.

Adding directivity caused larger improvement in noise reduction, and to some extent, resolution, than different preprocessing methods. This is shown by the five largest axial resolution improvements occurring using the five directivity methods in the point acquisition and being significantly lower in all acquisitions except the multi-point acquisition, where, as suggested, the behavior is different due to the lateral signal ROI positioning. Even here, five of the highest six reductions in the axial resolution are from directivity methods, showing that directivity has a more significant effect.

The order of noise reduction is the same both with and without directivity. In decreasing order, it is: bandpass, unfiltered, SVD, wavelet denoising and EMD. Wavelet denoising had its best values for noise reduction, lateral resolution, and axial resolution only in the acquisition it was optimized for, the point acquisition, which suggests it is not generally optimized. This is supported by the noise reduction and lateral resolution becoming progressively worse with the increasing complexity of the signal, moving from the point to multi-point and finger acquisitions. Bandpass appeared to offer moderate improvements in noise reduction without much cost to the resolution and was the only method to outperform the unfiltered method consistently. Inconsistent results for lateral resolution were obtained using EMD and SVD. Distinctive artefacts are seen in the point acquisition (red markers, Figure 3D and 3E).

While this paper sought to provide a comparison of the common preprocessing methods and not the algorithm for deployment itself, we note the calculation time was, depending on the method, 7 to 43 s for a high-resolution (1200 × 1200) image. While this is below the speed required for real-time imaging, this could be achieved using parallelization and GPU acceleration which have previously yielded acceleration factors of 140-fold [24] and 125- to 1000-fold [25]. 

Deep learning methods were also used for preprocessing, such as the usage of convolutional neural networks (CNNs) [26] for artefact removal, the extension of bandwidth, and object detection using region-based CNNs [27,28,29] to separate signals from artefacts. While these could yield impressive results in terms of noise reduction, they are limited by the need for ground truth upon which to train, such as anatomical markers. This is typically difficult to obtain, resulting in the use of simulated data by a majority of studies which have shown poor translation to experimental data [18]. This ultimately limits the reliability of images for pathology and hence clinical diagnoses. As a black box, they generally are unable to show the paper trail of how the image was derived.

Furthermore, we note that this work was only focused on established preprocessing methods from a practical point of view demonstrated directly by experiments. The results may be affected by different experimental conditions such as laser fluence, transducer performance, target absorption, etc. Analytical models [30,31,32] may be helpful in predicting, verifying, and generalizing the results.

## 5. Conclusions

In conclusion, we tested combinations of classical signal preprocessing methods and directivity for 2D PA image reconstruction and found that directivity yields considerable improvements in both noise reduction and lateral resolution with the multi-segment transducer. With and without directivity, only bandpass filtering consistently yielded an improvement. Moreover, the advantages and disadvantages of other preprocessing methods were also investigated, which may be helpful in determining better preprocessing practices in PAT reconstruction.

## Figures and Tables

**Table 1:** Parameters used for each preprocessing method.

Preprocessing	Parameter	Value/Type
Bandpass filter	Filter type Bandwidth	FIR 90% of the center frequency
Wavelet denoising	Wavelet family	Daubechies
	Order	4th
	Denoising method	Universal threshold
	Threshold rule	Hard
	Noise estimation	Level-dependent
EMD	Number of components used	2
SVD	Number of components used	1
Sensor directivity	Angular FWHM	±13.5 degrees

**Table 2:** Noise reduction (CNR, higher means better), peak SNR (higher means better) and resolution (FWHM, lower means better) compared between preprocessing methods. Comparisons with the actual imaging phantom are listed in the brackets under the FWHM columns.

			Point				Multi-point				Finger	
Method	CNR	Peak SNR	FWHM_x_ (μm)	FWHM_z_ (μm)	CNR	Peak SNR	FWHM_x_ (μm)	FWHM_z_ (μm)	CNR	Peak SNR	FWHM_x_ (μm)	FWHM_z_ (μm)
Target	-	-	150	150	-	-	150	150	-	-	-	-
Unfiltered	34	698	552.1 (368%)	780.3 (520%)	13.2	185	537.6 (358%)	783.2 (522%)	16.9	79	533.3	767.1
Bandpass	38.3	783	552.2 (368%)	780.5 (520%)	13.2	190	538.1 (359%)	786.2 (524%)	17.5	80	533.1	766.8
Wavelet	31.3	723	586.5 (391%)	777.5 (518%)	8	168	537.5 (358%)	778.1 (519%)	12.9	67	534.5	770.2
EMD	33.5	564	574.7 (383%)	781.7 (521%)	13.2	115	537.6 (358%)	781.2 (521%)	16.5	61	532.4	767.6
SVD	44.6	678	539.4 (360%)	778.9 (519%)	17.9	184	537.3 (358%)	803.7 (536%)	28.7	73	532.8	763.3
Unfiltered+ directivity	52.4	779	540.1 (360%)	779.5 (520%)	17.7	221	538.1 (359%)	813.6 (542%)	27.7	113	532.4	766.1
Bandpass+ directivity	46	917	539.3 (360%)	778.7 (519%)	15.7	226	537.2 (358%)	834.3 (556%)	25.7	106	532.9	766.6
Wavelet+ directivity	44.9	796	538.8 (359%)	780.5 (520%)	17.5	199	537.2 (358%)	787 (525%)	26.4	99	532.4	765.3
EMD+directivity	34	615	552.1 (368%)	780.3 (520%)	13.2	136	537.6 (358%)	783.2 (522%)	16.9	92	533.3	767.1
SVD+directivity	38.3	768	552.2 (368%)	780.5 (520%)	13.2	218	538.1 (359%)	786.2 (524%)	17.5	104	533.1	766.8

**Figure A1:**
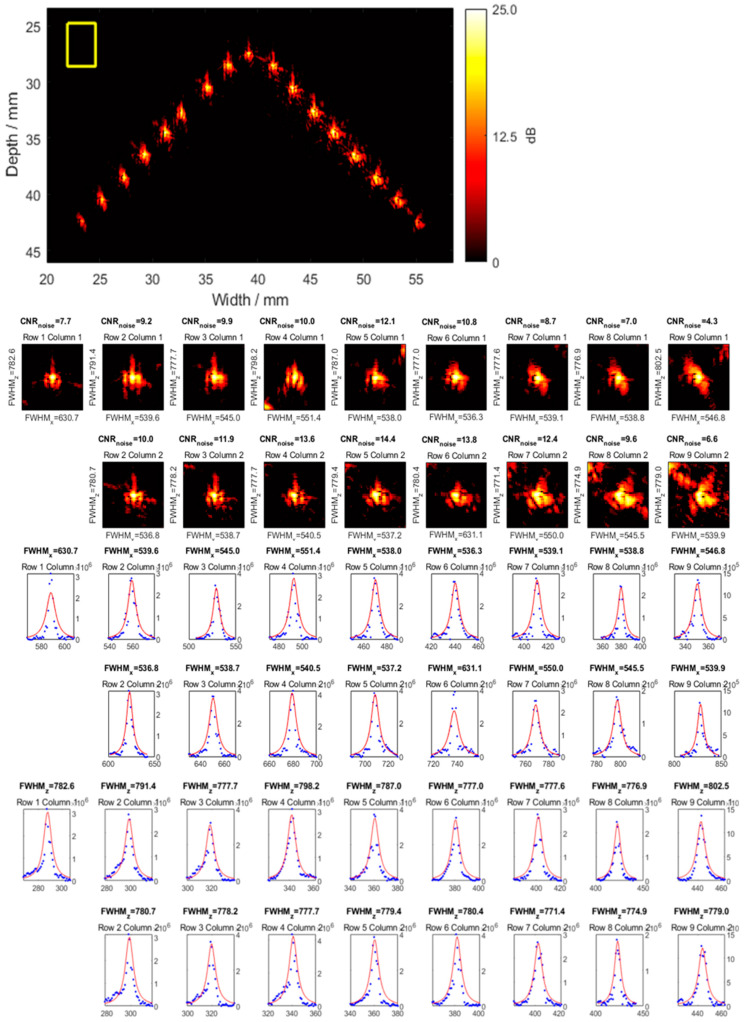
CNR, FWHMx, and FWHMz calculated for each of the 17 points in the multi-point acquisition.

## Data Availability

Data will be provided upon reasonable request.

## Appendix A

### Appendix A.1. Optimization of Wavelet Denoising

Wavelet denoising parameters (wavelet family, order, denoising method, threshold rule, and noise estimation) were optimized by the maximization of the CNR and the minimization of FWHM_x_ and FWHM_z_ on the point acquisition alone. Image processing was carried out using the default unfiltered method without directivity.

### Appendix A.2. Selection of the Wavelet Family and Order

Each wavelet family available in MATLAB was compared (Table A1), namely *coif* (Coiflets), *fk* (Fejer-Korovkin), *db* (Daubechies), *sym* (Symlet), and *haar* (Haar). For all except *haar*, the wavelet orders also needed to be specified. Starting from the lowest order, the orders were tested until the maximum order specifiable was reached, or the CNR was observed to decrease monotonically for at least two orders.

Default settings were used for the other required parameters: “DenoisingMethod” was set to “universal threshold”, “ThresholdRule” was set to “soft”, and “NoiseEstimation” was set to “level-dependent”. However, these settings were optimized in the following two stages.

**Table A1:** Comparison of the wavelet families and orders.

Family and Order Comparison									
Family	Order	CNR	Rank	FWHMx	Rank	FWHMz	Rank	Average Rank	Rank
coif	1	34.27	13	552.1	1	779.8	21	11.7	13
	2	34.74	10	553.07	10	779.54	11	10.3	11
	3	36.28	2	553.98	13	779.43	6	7.0	2
	4	36.03	3	554.46	15	779.46	7	8.3	6
	5	36.84	1	555.25	19	779.38	4	8.0	4
fk	4	33.61	20	554.25	14	779.76	20	18.0	21
	6	33.18	21	555.69	20	779.46	7	16.0	20
	8	34.83	8	553.61	11	779.39	5	8.0	4
db	1	33.65	17	552.56	6	779.68	17	13.3	15
	2	33.73	15	554.86	16	779.56	12	14.3	18
	3	34.36	11	552.51	4	779.67	15	10.0	9
	4	35.42	5	552.38	2	779.35	3	3.3	1
	5	35.15	7	553.63	12	779.2	2	7.0	2
	6	35.19	6	555.06	18	779.16	1	8.3	6
sym	1	33.65	17	552.56	6	779.68	17	13.3	15
	2	33.73	15	554.86	16	779.56	12	14.3	18
	3	34.36	11	552.51	4	779.67	15	10.0	9
	4	34.24	14	552.47	3	779.59	14	10.3	11
	5	35.79	4	556.38	21	779.49	10	11.7	13
	6	34.78	9	552.77	9	779.46	7	8.3	6
haar		33.65	17	552.56	6	779.68	17	13.3	15

The Daubechies family, with the fourth order, was selected as this was the highest-ranked.

Next, all permutations of “DenoisingMethod” and “ThresholdRule” were compared to find the best “ThresholdRule” in each “DenoisingMethod” (Table A2). Since using the absolute rank produced too many ties, the rank of the average % behind the best was used.

**Table A2:** Comparison of the permutations of “DenoisingMethod” and “ThresholdRule”.

db4 DenoisingMethod and ThresholdRule Comparison									
DenoisingMethod	ThresholdRule	CNR	Rank	FWHMx	Rank	FWHMz	Rank	Average % behind Best	Rank
UniversalThreshold	Soft	35.42	11	552.38	12	779.35	1	1.14%	2
	Hard	35.48	10	550.52	1	780.25	5	1.15%	3
Bayes	Median	36.19	5	550.68	3	780.34	8	1.85%	7
	Mean	36.22	4	550.84	5	780.33	7	1.89%	9
	Soft	36.15	6	551.67	9	779.86	3	1.85%	8
	Hard	36.28	3	550.72	4	780.43	12	1.94%	10
Minimax	Soft	35.9	8	551.7	10	779.65	2	1.60%	5
	Hard	36.03	7	550.66	2	780.41	11	1.69%	6
FDR	Hard	35.51	9	550.97	6	780.37	9	1.21%	4
BlockJS	James-Stein	34.34	12	552.09	11	780.27	6	0.13%	1
SURE	Soft	36.48	1	551.22	8	780.17	4	2.15%	12
	Hard	36.29	2	551.12	7	780.4	10	1.97%	11

Finally, each of these combinations was compared using both level-dependent and level-independent noise estimation (Table A3). The highest-ranked combination was used. In total, this was Daubechies fourth order, universal threshold, hard thresholding, and level-dependent noise estimation.

**Table A3:** Comparison of noise estimation with and without level dependence.

db4 NoiseEstimation Comparison									
DenoisingMethod/ ThresholdRule	NoiseEstimation	CNR	Rank	FWHM_x_	Rank	FWHM_z_	Rank	Average Rank	Rank
UniversalThreshold/hard	LevelDependent	35.48	5	550.52	1	780.25	2	2.67	1
	LevelIndependent	34.09	4	552.22	10	780.29	3	5.67	6
Bayes/median	LevelDependent	36.15	9	551.67	5	779.86	1	5.00	4
	LevelIndependent	34.25	5	552.12	8	780.31	4	5.67	6
Minimax/hard	LevelDependent	36.03	8	550.66	2	780.41	10	6.67	9
	LevelIndependent	33.99	2	552.11	7	780.32	5	4.67	2
FDR/hard	LevelDependent	35.51	7	550.97	3	780.37	8	6.00	8
	LevelIndependent	33.98	1	552.12	8	780.32	5	4.67	2
SURE/hard	LevelDependent	36.29	10	551.12	4	780.4	9	7.67	10
	LevelIndependent	34	3	552.07	6	780.33	7	5.33	5

## Appendix B

### Selection of the Optimal Row from the Multi-Point Acquisition

For each of the 17 points in the multi-point acquisition, the CNR, FWHM_x_, and FWHM_z_ were individually calculated (Figure A1).

The results (Table A4) were organized into rows, with the first row consisting of one point, followed by eight rows of two points. The average CNR, FWHM_x_, and FWHM_z_ were calculated for each row. It was decided to use row five because it had the best values for the CNR and FWHM_x_.

**Table A4:** Comparison of CNR and resolution for each row in the multi-point acquisition.

	CNR (μm)		FWHM_x_ (μm)		FWHM_z_ (μm)	
Row	µ	σ	µ	σ	µ	σ
1	7.71	N/A	630.71	N/A	782.56	N/A
2	9.6	0.59	538.2	1.98	786.06	7.58
3	10.89	1.42	541.88	4.47	777.97	0.36
4	11.78	2.5	545.96	7.71	787.91	14.5
5	13.25	1.61	537.6	0.53	783.23	5.37
6	12.28	2.11	583.72	67.04	778.73	2.39
7	10.52	2.62	544.54	7.74	774.52	4.41
8	8.33	1.84	542.12	4.74	775.93	1.43
9	5.42	1.63	543.37	4.89	790.78	16.62
